# Bangpungtongsung-san alleviates depressive-like behavior and metabolic disturbances in high-fat diet-induced obesity: mechanisms involving inflammation, CREB/BDNF signaling, and NMDA receptor modulation

**DOI:** 10.3389/fphar.2025.1565592

**Published:** 2025-10-30

**Authors:** Bo-Ram Lee, No Soo Kim, Ui-Jin Bae, Yujin Choi, Changsop Yang, Mi Young Lee

**Affiliations:** ^1^ KM Convergence Research Division, Korea Institute of Oriental Medicine, Daejeon, Republic of Korea; ^2^ KM Science Research Division, Korea Institute of Oriental Medicine, Daejeon, Republic of Korea

**Keywords:** Bangpungtongsung-san, depression-like behavior, high fat diet-induced obesity, CREB/BDNF signaling, NMDA receptor, comorbidity

## Abstract

**Introduction:**

Bangpungtongsung-san (BTS) is a traditional multi-herb preparation prescribed for obesity, but its role in obesity-associated depression remains unclear. We evaluated whether BTS alleviates depressive-like behaviors in high-fat diet (HFD) induced obese mice and elucidated the underlying mechanisms of its antidepressant potential.

**Methods:**

Male C57BL/6N mice were randomized to normal diet (ND) or continuous HFD and maintained for 10 weeks. Throughout this period, mice were orally treated with BTS (30, 100, or 300 mg/kg), fluoxetine (FXT), simvastatin (SIM), or vehicle under identical chronic regimens. Body weight was monitored weekly. At week 10, metabolic parameters (blood glucose, plasma total cholesterol, triglycerides, HDL-C, and leptin) and depressive-like behaviors (tail suspension test and forced swimming test) were assessed. Subsequently, mechanistic analyses were performed to determine the effects of BTS on systemic and brain inflammatory responses, BDNF signaling, NMDAR expression, and serotonin (5-HT) signaling (Ido1, Tph2, and SERT) in the prefrontal cortex (PFC) and hippocampus (HPC).

**Results:**

A 10-week continuous HFD feeding produced robust weight gain, hyperglycemia, and elevated levels of total cholesterol (TCHO), triglycerides (TG), HDL-C, and leptin. Oral BTS treatment attenuated body weight gain and reversed these HFD-induced metabolic abnormalities (TCHO, TG, HDL-C, and leptin) in blood. Behaviorally, BTS-treated mice exhibited reduced immobility time compared to HFD group, indicating antidepressant-like effects. Mechanistically, BTS reduced systemic and brain pro-inflammatory cytokines (IL-1β and TNF-α) and normalized hippocampal GluN1/GluN2A/GluN2B protein levels together with BDNF expression restoration. BTS also elevated whole-brain 5-HT and tended to regulate SERT expression in HPC, supporting the enhanced synaptic 5-HT availability. Under identical chronic oral conditions, FXT showed partial antidepressant efficacy with minimal metabolic benefits, whereas SIM exhibited moderate metabolic improvements with limited behavioral effects. Comparatively, BTS provided superior therapeutic outcomes across both behavioral and metabolic parameters.

**Conclusion:**

BTS ameliorated depression-like behaviors and metabolic dysfunction in HFD-induced obesity through coordinated modulation of inflammation, BDNF signaling, NMDAR expression, and 5-HT neurotransmission in the HPC. These findings support BTS as a promising multi-target candidate for treating comorbid depression and obesity.

## 1 Introduction

According to the World Health Organization, more than 650 million adults, 340 million adolescents, and 39 million children are diagnosed with obesity, and at least 2.8 million people die of overweight- or obesity-related conditions each year globally. Obesity is a non-communicable condition associated with a wide range of diseases and frequent comorbidity with mental disorders, such as anxiety- and depression-like behaviors (Iacovides and Siamouli, 2008; Rosas et al., 2020). People with obesity have lower self-esteem, feelings of guilt, and social anxiety, which in turn lead to eating disorders (Albohn-Kuhne and Rief, 2011). A national health and nutritional survey in the U.S. showed an increased prevalence of obesity in adults with depression, observed in women and men of all age groups over 60 (Pratt and Brody, 2014). Supporting these clinical observations, several preclinical studies have demonstrated anxiety- and depression-associated behaviors in mice with high-fat diet (HFD)-induced obesity (Yamada et al., 2011; Vagena et al., 2019), as well as in genetically modified mice with metabolic disorders (Collin et al., 2000; Sharma et al., 2010). In particular, obesity-related depressive-like behaviors have been linked to an inflammatory transcriptional factor, which disrupt glutamatergic signaling in the hippocampus (HPC) (Li et al., 2022). Furthermore, HFD-induced obesity caused glucose metabolic dysfunction and depressive-like behaviors, in part by reducing hippocampal astrocyte activation (Lam et al., 2021). Therefore, these findings indicate that obesity is not merely a metabolic disorder but a condition that substantially increases the risk of mental disorders through shared biological mechanisms, and their comorbidity is of great value to study.

Inflammation and hormone dysregulation are known to play crucial roles in obesity and mood disorder comorbidity (Jarolimova et al., 2013; Castanon et al., 2014). Additionally, disturbances in the brain serotonergic system are important contributors to the comorbidity of obesity and mood disorders. Experimental studies using HFD models have demonstrated these disturbances, showing that peripheral and central serotonergic changes co-occurred with mood disorders. Specifically, 5-HT levels in plasma and hippocampal 5-HT transporter expression were dysregulated with accompanying depressive behaviors (Kim et al., 2013; Hersey et al., 2021). Beyond metabolic disease models, genetic manipulations have shown that central 5-HT insufficiency promotes hyperphagia, metabolic disorders, and impairs mood regulation (Conde et al., 2023). In particular, mice with brain 5-HT deficiency caused by genetic modifications suffered from a binge-like eating disorder and abolished hypothalamic neuronal activation (Liu et al., 2021; Karth et al., 2022). Moreover, serotonin transporter deficiency has been shown to exacerbate adipose tissue inflammation in obese mice, suggesting that serotonin dysregulation is directly related to obesity-related metabolic disturbances (Hoch et al., 2023). Furthermore, a clinical investigation demonstrated that altered peripheral serotonin signaling in adipose tissue contributes to both obesity and insulin resistance (Park et al., 2025). Taken together, these findings support the hypothesis that serotonergic dysfunction is a key mechanistic pathway linking metabolic dysfunction and mood disorders.

Chronic antidepressant use causes various adverse effects, such as diarrhea, dyspepsia, nausea, sexual dysfunction, and sleep disturbance (Bet et al., 2013; Read et al., 2014). In particular, 65.3% of patients experience weight gain as an adverse effect of antidepressant medications (Cartwright et al., 2016). Moreover, preclinical and clinical studies have demonstrated that metabolic disturbances may contribute to poor responses to standard antidepressant treatment. For example, long-term administration of escitalopram, a selective 5-HT reuptake inhibitor (SSRI), did not improve anxiety- and depression-like behaviors in HFD-induced obese mice (Zemdegs et al., 2016). Additionally, the antidepressant SSRI effect was less pronounced in streptozotocin-induced diabetic mice than in non-diabetic mice (Kamei et al., 2003). In clinical settings, the therapeutic effect of antidepressants is insufficient in patients with depression and diabetes (Anderson et al., 2010) or obesity (Kloiber et al., 2007; Oskooilar et al., 2009). These results indicate that the pharmacological efficacy of first-line antidepressant therapy is insufficient or at least limited in patients with depression suffering from metabolic diseases. These clinical demands prompted us to search for novel effective therapeutics with minimal adverse effects to treat patients with depression and metabolic diseases.

Given the limitations of current treatment on comorbid depression in metabolic disorders, herbal medicines have garnered increasing interest as potential therapeutic options for treating both obesity and depression, as reported by recent clinical studies (Chandrasekaran et al., 2012). Bangpungtongsung-san (BTS; Fang-feng-tong-sheng-san in Chinese or Bofutsushosan in Japanese) is a traditional herbal medicinal preparation that has long been prescribed in East Asia for obesity (Uneda et al., 2022; Oh et al., 2020). It was first introduced in the classical Chinese herbal book, *Formulas from the Discussion Illuminating the Yellow Emperor’s Basic Questions* (*Hugang Di Su Wen Xuan Ming Lun Fang*) in 1172 (Liu et al., 2004). BTS has been utilized for managing ailments such as high fever, vertigo, tinnitus, ear congestion, nasal problems, skin diseases, constipation, and pulse problems (Lee et al., 2005; Lee C. W. et al., 2012). Clinical evidence supports the efficacy of BTS in reducing body weight and improving obesity-related parameters. In a randomized controlled trial involving patients with obesity-related hypertension, 24-week administration of BTS significantly reduced body weight and BMI compared to the control group (Azushima et al., 2015). A meta-analysis of randomized controlled clinical studies revealed that BTS could potentially improve the body mass index of obese participants without serious adverse events (Uneda et al., 2022). In preclinical studies, BTS decreased total cholesterol (TCHO) blood levels and liver lipid levels (Shin and Song, 1997) and downregulated obesity-related gene expression in HFD-induced obese mice (Hwang et al., 2006). In addition, BTS could improve HFD-induced insulin resistance by regulating lipid metabolism, suggesting that BTS could be an effective therapeutic option to manage metabolic diseases (Seo et al., 2018).

Our previous preclinical study demonstrated the antidepressant potential of BTS in mice with reserpine-induced depression via reciprocal regulation of protein expression in the brain associated with neurogenesis and neuroinflammation (Park et al., 2020). Based on the common clinical use of BTS for obesity-related disorders, we hypothesized that BTS might have therapeutic potential for obesity-associated depression. In the present study, we evaluated the antidepressant potential of BTS in HFD-induced obese mice. First, we compared the effect of HFD on depression-like behaviors and blood parameters in mice through continuous vs. intermittent HFD (IHFD) feeding. Second, we evaluated the antidepressant potential of BTS by assessing metabolic parameters, as well as depression-like behaviors in mice with continuous HFD-induced obesity. Finally, we explored potential mechanisms by analyzing neurochemical changes in the brains of experimental mice, focusing on pathways implicated in both obesity and depression, including inflammation, neurotrophic signaling, and neurotransmitter systems. Through this comprehensive investigation, we aim to provide a detailed understanding of how BTS might simultaneously address obesity and depression, potentially offering a novel therapeutic approach for this complex comorbidity.

## 2 Materials and methods

### 2.1 Preparation of herbal extract

BTS extract was prepared according to our previous report (Park et al., 2020). The herbal components of BTS extract and their composition ratios are presented in Table 1. In brief, all herbal ingredients were mixed according to their specified ratios and extracted in boiling water for 3 h. Then, the extracted solution was filtered and concentrated under a vacuum to obtain a final dried extract with a yield of 12.97% (w/w). The dried extract powder was stored at −80 °C or dissolved in phosphate-buffered saline (PBS, pH 7.4) right before use. BTS was standardized by comparing its retention time and UV spectrum with those of each reference standard (gallic acid, geniposide, albiflorin, paeoniflorin, liquiritin apioside, liquiritin, nodakenin, benzoic acid, baicalin, wogonoside, and glycyrrhizin) via HPLC-PDA (Park et al., 2020). The HPLC-PDA profile of BTS, including the retention times and UV spectra of BTS, were reported in our prior study (Park et al., 2020; Supplementary Figure S1). The quantified contents of 11 marker compounds in BTS extract were listed in Supplementary Table S1.

**TABLE 1:** Composition of bangpuntongsung-san (BTS) extract.

Herbal name	Botanical name [family]	Part used	Ratio
*Angelica gigas*	*Angelica gigas* Nakai [Apiaceae]	Root	1
*Paeonia lactiflora*	*Paeonia lactiflora* Pall. [Paeoniaceae]	Root	1
*Cnidium officinale*	*Ligusticum officinale* (Makino) Kitag. [Apiaceae]	Rhizome	1
*Forsythia viridissima*	*Forsythia viridissima* Lindl. [Oleaceae]	Fruit	1
*Mentha arvensis*	*Mentha arvensis* L. [Lamiaceae]	Shoot	1
*Saposhnikovia divaricata*	*Saposhnikovia divaricata* (Turcz.) Schischk. [Apiaceae]	Root	1
*Ephedra sinica*	*Ephedra sinica* Stapf [Ephedraceae]	Stem	1
*Rheum undulatum*	*Rheum undulatum* L. [Polygonaceae]	Root and rhizome	1.25
*Natrii sulfas*	-	-	1.25
*Platycodon grandiflorum*	*Platycodon grandiflorus* (Jacq.) A.DC. [Campanulaceae]	Root	1.68
*Scutellaria baicalensis*	*Scutellaria baicalensis* Georgi [Lamiaceae]	Root	1.68
Gypsum	-	-	2.5
*Zingiber officinale*	*Zingiber officinale* Roscoe [Zingiberaceae]	Rhizoma	1
*Gardenia jasminoides*	*Gardenia jasminoides* J. Ellis [Rubiaceae]	Fruit	1
*Schizonepeta tenuifolia*	*Nepeta tenuifolia* Benth. [Lamiaceae]	Flower stalk	1
*Atractylodes japonica*	*Atractylodes lancea* (Thunb.) DC. [Asteraceae]	Rhizome	1.68
*Glycyrrhiza uralensis*	*Glycyrrhiza uralensis Fisch*. ex DC. [Fabaceae]	Root and rhizome	1.68
Talcum	-	-	1.68

### 2.2 Animal

Male C57BL/6N mice (6-week-old, 20–23 g) were supplied by Saeronbio (Uiwang-si, Republic of Korea). All animals were housed in a polycarbonate cage (maximum five mice per cage) and acclimated for 1 week in a specific pathogen-free laboratory animal care facility under standard conditions: 22 °C ± 2 °C room temperature, 45% ± 10% relative humidity, 12/12 h light/dark cycle. Animals were allowed free access to food and tap water.

Each group was initially allocated n = 10 mice by block randomization (baseline body weight). During the 10-week regimen, occasional model-related attrition in HFD-fed mice (spontaneous death or euthanasia under humane endpoints) was recorded. The animal was excluded from that assay. No outcome-based exclusion was permitted. Final per-assay sample size was reported in the figure and legends. All animal experimental procedures were conducted in accordance with the guidelines for the Care and Use of Laboratory Animals of the Ministry of Food and Drug Safety, Republic of Korea. All procedures were reviewed and approved by the Institutional Animal Care and Use Committee of the Korea Institute of Oriental Medicine (Protocol # 20–085 and 21–028).

### 2.3 HFD-induced obesity-depression model

Increasing evidence has demonstrated that obesity induced by chronic calorie-dense HFD intake is associated with depression-like behaviors in experimental rodents (Boitard et al., 2014; Arcego et al., 2018; Vagena et al., 2019). We used three different diet programs to investigate the effect of obesity on depression-like behavior in experimental animals (n = 10 per group, a total of 30 mice): normal diet group (ND, 12.4% kcal/fat, 24,5% kcal/protein, 63.1% kcal/carbohydrate, total 394 kcal/100 g, Purina laboratory rodent diet #38057, Purina Korea Inc., Seoul, Republic of Korea), HFD group (60% kcal/fat, 20% kcal/protein, 20% kcal/carbohydrate, total 524 kcal/100 g, D12492, Research Diets Inc., New Brunswick, NU, United States), and IHFD group (alternating 1-week ND/1-week HFD). The HFD calorie value was 524 kcal/100 g, 33% calorie-denser than ND (394 kcal/100 g). The mice were fed according to the dietary schedule for 10 weeks to induce obesity (Supplementary Figure S1). Body weight was recorded once a week throughout the experiment. After a 10-week diet program, behavioral assessments were conducted to assess depression- and anxiety-like despair of animals under stressful conditions. We confirmed that HFD mice exhibited (ⅰ) weight gain and increased metabolic abnormalities and (ⅱ) increased immobility (TST and FST) and anxious behaviors (increased total distance moved and the number of center crossing in OFT) compared to ND. These results indicated that depression-like behaviors could be developed by chronic and continuous 10-week HFD intake in adult male C57BL/6N mice. Full validation outcomes are reported in Supplementary Figure S1.

### 2.4 Experimental design

**FIGURE 1:**
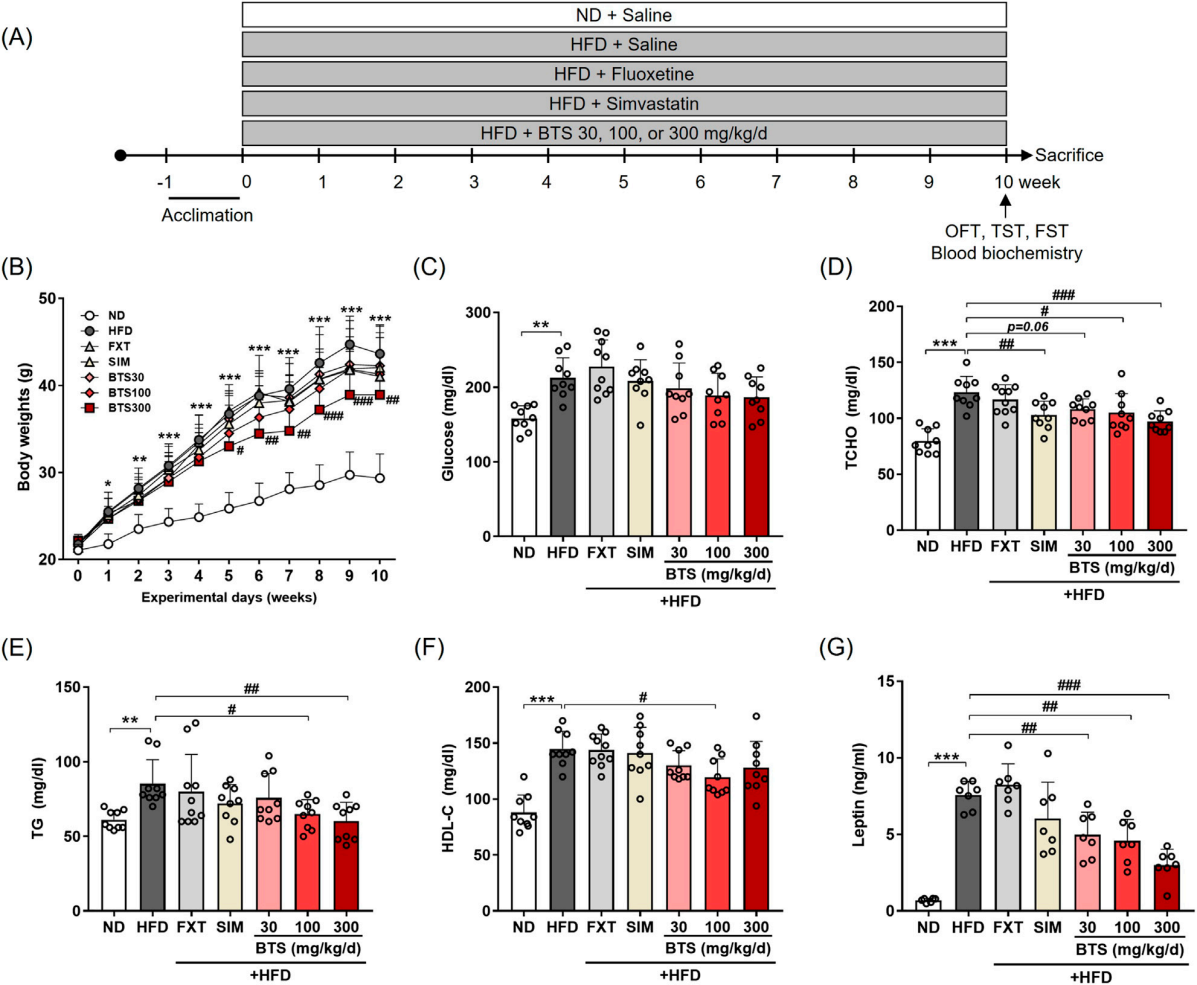
Effects of BTS on body weight and metabolic states in mice with HFD-induced obesity. Mice were fed ND or HFD for 10 weeks in the presence of vehicle or BTS (30, 100, or 300 mg/kg) on a daily basis. FXT and SIM were also administered in parallel as positive control drugs for depression and obesity, respectively. **(A)** Schematic diagram of diet schedule and behavior tests. **(B)** Body weights (n = 9–10/group) were recorded every week throughout the experimental period. The values are presented as mean ± SD; two-way ANOVA + Dunnett’s *post hoc*. (control = HFD). Fasting glucose **(C)**, TCHO **(D)**, TG **(E)**, HDL-C **(F)**, and leptin **(G)** were analyzed from plasma collected at the experimental endpoint (n = 6–10). The values are presented as mean ± SD with individual data points; one-way ANOVA + Dunnett’s *post hoc* (control = HFD). ^*^ *p* < 0.05, ^**^ *p* < 0.01, and ^***^ *p* < 0.001 vs. ND; ^#^ *p* < 0.05, ^##^ *p* < 0.01, and ^###^ *p* < 0.001 vs. HFD.

In the main study to investigate the antidepressant potential of BTS, mice were randomly divided into seven groups (n = 10 per group, a total of 70 mice): mice were fed ND (group 1) or HFD (groups 2–7) for 10 weeks. During the 10-week obesity induction period, groups 1 and group 2 received 0.9% (w/v) saline as a vehicle. Groups 3 and 4 received fluoxetine (FXT, 20 mg/kg/day, #F132, Sigma, St Louis, MO, United States) and simvastatin (SIM, 9 mg/kg/day, #S6196, Sigma) via oral gavage as antidepressant and hypolipidemic control drugs, respectively. Groups 5–7 received BTS at doses of 30, 100, and 300 mg/kg/day, respectively. The BTS doses were chosen according to our previous study, showing its antidepressant potential in both *in vivo* and *in vitro* models (Park et al., 2020). All drugs, including saline, were administered via oral gavage. Body weight was recorded once a week throughout the experiment. The experimental schemes used in the main study are shown in Figure 1A.

### 2.5 Behavioral assessment

An open-field test (OFT) measuring spontaneous activity and emotional responsiveness (Budni et al., 2007) was performed as described in our previous study (Park et al., 2020). Briefly, mice were individually placed in the center of an acrylic open field arena (30 × 30 cm), and their free exploratory behaviors were video-recorded for 10 min. Animal behavioral parameters, such as total distance traveled, time spent in the center zone, and number of four-paw center crossings, were analyzed using video tracking software (EthoVision XT 9.0, Noldus Information Technology, Wageningen, Netherlands). Depression-related behavioral despair was measured using the tail suspension test (TST) and forced swimming test (FST) as previously described (Pan et al., 2019). TST was performed in both visually and acoustically isolated areas. The mice were suspended 50 cm above the floor by applying adhesive tape approximately 1 cm from the tail tip. The movement of individual mice was video-recorded for 6 min, and the total immobility time of mice showing passive or floating behaviors in the water was analyzed during the last 4 min of the test session using a video tracking software (SMART 3.0, Panlab S.I., Barcelona, Spain). During the FST, all mice were subjected to forced pre-swim training for 15 min. On the following day, mice were separately placed in an acrylic cylinder (45 cm height, 20 cm diameter) containing tap water (25 °C ± 2 °C, 25 cm depth) and then allowed to swim freely for 6 min. Considering the mouse test burden, behavior was assessed in the order of OFT → TST → FST, with at least 6 h intervals between each experiment.

### 2.6 Blood, plasma, and tissue collection

After behavioral assessment, the mice were deeply anesthetized via intraperitoneal injection of fresh 2% (w/v) avertin (240 mg/kg, Sigma, #48402) dissolved in tert-amyl alcohol (Sigma, #152463). Whole blood was collected in a heparinized tube via cardiac puncture, mixed on a roller for 30 min, and centrifuged at 1,000 × g for 15 min at 4 °C. The clear yellow plasma was recovered, aliquoted, and stored at −80 °C until use. Whole brains or sub-brain prefrontal cortex (PFC)/HPC tissues were harvested from half of the mice in each group, immediately snap-frozen in a liquid nitrogen, and stored at −80 °C until use.

### 2.7 Western blotting

Total protein extract was isolated from frozen PFC or HPC tissues using a PreCellys mechanical homogenizer (Bertin Instruments, Montigny-le-Bretonneux, France) in RIPA buffer supplemented with Halt™ protease and phosphatase inhibitors (#78444, Thermo Fisher Scientific, Rockford, IL, United States). A total of 20 μg protein lysate was subjected to SDS-PAGE and transferred to polyvinylidene difluoride membranes (Bio-Rad, Hercules, CA, United States). The membranes were blocked with a 5% skim milk for 1 h at room temperature and then incubated with a blocking solution containing primary antibodies overnight at 4 °C. The primary antibodies used for Western blotting were as follows: β-actin (sc-47778, clone C4, Santa Cruz, Dallas, TX, United States), brain-derived neurotrophic factor (BDNF, sc-65514, clone 5H8, Santa Cruz), cAMP response element-binding protein (CREB, #9197, clone 48H2, Cell Signaling Technology, Danvers, MA, United States), phospho-CREB (pCREB, #9198, clone 87G3, Cell Signaling Technology), N-methyl-D-aspartate (NMDA) receptor 1 [GluN1, ab109182, clone EPR2481 (2); Abcam, Cambridge, United Kingdom], GluN2A [ab124913, clone EPR2465 (2), Abcam], and GluN2B (ab65783, Abcam). After washing 3 times for 10 min each in TRIS-buffered saline containing 0.1% Tween 20, the membranes were incubated with appropriate secondary antibodies conjugated with horseradish peroxidase (Enzo Life Sciences, NY, United States) for 1 h at room temperature. The proteins of interest were detected by incubating the membrane in a chemiluminescent substrate solution (#1705062, Bio-Rad), and band intensities were quantified using an ImageQuant™ LAS-4000 image analyzer (GE Healthcare Life Science, Pittsburgh, PA, United States).

### 2.8 RNA isolation and quantitative real-time chain reaction (qPCR)

Total RNA was prepared from frozen PFC or HPC tissues using an Easy-Spin™ total RNA extraction kit (#17221, iNtRON Biotechnology, Seongnam, Republic of Korea). First-strand cDNA was synthesized from 2 µg of total RNA using a High-Capacity cDNA Reverse Transcription Kit (#4368814, Thermo Fisher Scientific) according to the manufacturer’s instructions. Gene-specific forward and reverse primer pairs were designed using PrimerBank (https://pga.mgh.harvard.edu/primerbank), and their sequences are summarized in Table 2 qPCR was carried out in a CFX96™ real-time PCR system (Bio-Rad) in a 10 μL reaction volume containing 250 nM of each primer and Power SYBR™ Green PCR master mix (#4367659, Thermo Fisher Scientific). The target genes were normalized to the housekeeping gene glyceraldehyde 3-phosphate dehydrogenase (GAPDH), and their relative gene expression in the control group was determined by the 2^−ΔΔCT^ method.

**TABLE 2:** Primer sequences used for qPCR and accession number of target gene.

Gene	Forward (5'→3′)	Reverse (5'→3′)	Amplicon (bp)	Access. No.
*Gapdh*	CGT​CCC​GTA​GAC​AAA​ATG​GT	TTG​ATG​GCA​ACA​ATC​TCC​AC	110	NM_008084
*GluN1*	AGA​GCC​CGA​CCC​TAA​AAA​GAA	CCC​TCC​TCC​CTC​TCA​ATA​GC	171	NM_008169
*GluN2a*	TGA​TGA​ACC​GCA​CTG​ACC​CTA	TGG​GGA​TGA​AAG​TCT​GTG​AGG	153	NM_008170
*GluN2b*	GCC​ATG​AAC​GAG​ACT​GAC​CC	GCT​TCC​TGG​TCC​GTG​TCA​TC	107	NM_008171
*Ido1*	CCT​GGT​TTT​GAG​GTT​TTC​GTG​TA	AAG​GTT​TCA​GCA​TTA​AGA​AGG​TTG	98	NM_001293690
*Il1β*	GCT​GAA​AGC​TCT​CCA​CCT​CA	AGG​CCA​CAG​GTA​TTT​TGT​CG	104	NM_008361
*Il6*	GAG​GAT​ACC​ACT​CCC​AAC​AGA​CC	AAG​TGC​ATC​ATC​GTT​GTT​CAT​ACA	141	NM_031168
*Sert*	GCT​CAT​CTT​CAC​CAT​TAT​CTA​CTT​C	AGT​TTC​TGC​CAG​TTG​GGT​TTC	180	NM_010484
*Tnfα*	AGA​CCC​TCA​CAC​TCA​GAT​CAT​CTT​C	CCA​CTT​GGT​GGT​TTG​CTA​CGA	78	NM_013693
*TpH2*	TCT​ACA​CCC​CGG​AAC​CAG​ATA​CA	CTC​CCA​GAG​ACG​CTA​AGC​CTA​TC	111	NM_173391

### 2.9 Enzyme-linked immunosorbent assay (ELISA)

Frozen whole brains were homogenized twice in 1 mL ice-cold PBS using a PreCellys mechanical homogenizer at 4,000 rpm and then diluted with 4 mL ice-cold PBS. After two freezing-thawing cycles, the homogenate was centrifuged at 10,000 rpm for 10 min at 4 °C. The clear lysate was recovered and used for monoamine ELISA assays. Brain lysate 5-HT (ab133053, Abcam), dopamine (DA) (CSB-E08661m, Cusabio, Huston, TX, United States), and norepinephrine (NE) (CSB-E07870m, Cusabio) in brain lysates or leptin (CSB-E04650m, Cusabio), tumor necrosis factor-alpha (TNF-α) (CSB-E04741m, Cusabio), interleukin 1-beta (IL-1β) (CSB-E08054m, (Cusabio), IL-6 (CSB-E04639m, Cusabio), and corticosterone (#501320, Cayman Chemical, Ann Arbor, MI, United States) in plasma were quantified using commercial ELISA kits according to the manufacturer’s guidelines. Color development was monitored and measured at 405 nm using a SpectraMax3 microplate reader (Molecular Devices, Sunnyvale, CA, United States).

### 2.10 Blood biochemical analyses

Metabolic states of the animals were determined by measuring blood glucose and plasma TCHO, triglyceride (TG), and high-density lipoprotein cholesterol (HDL-C). Blood glucose levels were determined using a CareSens N blood glucose monitoring system (i-Sense Inc., Seoul, Republic of Korea). The plasma levels of TG, TCHO, and HDL-C were measured on a Fuji Dri-Chem 7000i biochemistry automatic analyzer (Fujifilm, Tokyo, Japan) using dedicated DRI-CHEM slides: TG slide (#1650, Fujifilm), TCHO slide (#1450, Fujifilm), and HDL-C slide (#2650, Fujifilm), following the manufacturer’s instructions.

### 2.11 Statistical analysis

Changes in body weight were analyzed by two-way ANOVA. Other tests were performed using a one-way ANOVA. These data were analyzed with Dunnett’s test (control = HFD). All data were expressed as mean ± standard deviation (SD), and the mean differences were considered significant at *p* < 0.05.

## 3 Results

### 3.1 BTS attenuates HFD-induced obesity and metabolic disturbances

Before the main BTS study, we validated the model by comparing three diet programs (ND, HFD, IHFD; Supplementary Figure S1). After 10 weeks, HFD mice showed an increase in body weight versus ND. IHFD mice fluctuated with alternating diets and lost weight during ND weeks but ultimately gained more than ND. HFD also elevated blood glucose, plasma cholesterol (TCHO, TG, HDL-C) and leptin. IHFD displayed intermediate dysmetabolism higher than ND but generally lower than HFD, especially for glucose, TCHO, and leptin. Behaviorally, HFD increased immobility time in FST and decreased spontaneous exploration in OFT (reduced total movement and center crossing frequency) relative to ND, consistent with a depression-like phenotype. Taken together, these findings indicate that chronic, persistent HFD induced metabolic abnormalities and depression-like behaviors. Therefore, this study employed a chronic HFD program for subsequent study.

As BTS is traditionally prescribed to treat various diseases, including obesity (Lee M. Y. et al., 2012), we next investigated its effect on metabolic states in mice exposed to a chronic HFD for 10 weeks. BTS was co-administered with HFD in a dose-dependent manner (30–300 mg/kg/day, Figure 1A) throughout the diet schedule. For comparison, FXT or SIM was also co-administered with HFD in parallel as an antidepressant or a hypolipidemic control drug, whereas the HFD control mice received a saline vehicle. After 10 weeks, all groups fed HFD showed significant increases in body weight compared to ND mice irrespective of pharmaceutical interventions (Figure 1B). However, BTS treatment alleviated HFD-induced obesity, especially BTS 300 mg/kg showed a significant decrease in body weight compared to the HFD control mice from the 5^th^ week. In addition to ameliorating obesity, BTS could restore the HFD-induced metabolic disturbance in a dose-dependent manner (Figures 1C–G). BTS at 100 and 300 mg/kg significantly reduced TCHO and TG, and BTS 100 mg/kg group also decreased HDL-C. However, BTS treatment did not affect HFD-induced blood glucose elevation. Leptin, an energy balance hormone that downregulates food intake behavior (Mainardi et al., 2010), was also markedly elevated by HFD, but was dose-dependently reduced by BTS. As expected, SIM significantly reduced TCHO, whereas FXT did not regulate any noticeable metabolic changes in any parameters. A few studies have reported a risk of mild-to-severe diarrhea associated with chronic BTS treatment (Hioki et al., 2004; Shimada et al., 2008). In our study, however, the reduced body weight gain observed in mice with a high dose of BTS (300 mg/kg) was not considered to be due to BTS toxicity, as no toxicity-related signs, including diarrhea and reduced food intake, were observed throughout the experimental period (data not shown). These results indicated that BTS could improve obesity and metabolic disturbances induced by chronic HFD intake without any significant drug-related toxicity.

### 3.2 BTS ameliorates HFD-induced depression-like behaviors

**FIGURE 2:**
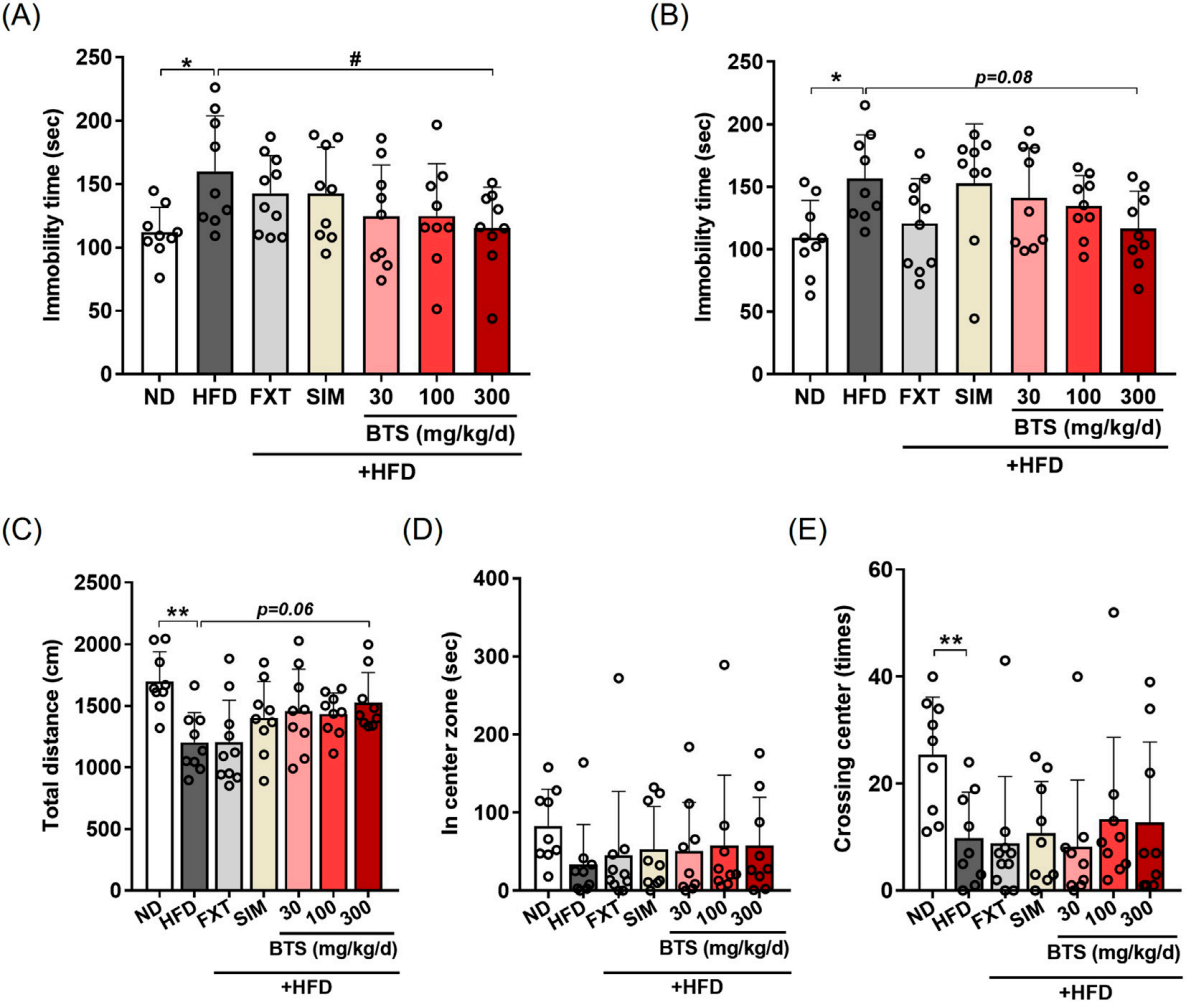
Effects of BTS on depressive behaviors in experimental mice with HFD-induced obesity. Mice were fed either ND or HFD for 10 weeks in the presence of vehicle, BTS (30, 100, or 300 mg/kg), FXT, or SIM on a daily basis. Immobility time in the TST **(A)**, FST **(B)**, total traveling distance **(C)**, time spent in the center zone **(D)**, and number of center crosses **(E)** in the OFT of individual mice were measured at the end of the study (n = 9–10). The values are presented as mean ± SD with individual data points; one-way ANOVA + Dunnett’s *post hoc* (control = HFD). ^*^ *p* < 0.05, and ^**^ *p* < 0.01 vs. ND; ^#^ *p* < 0.05 vs. HFD.

We hypothesized that if BTS could improve HFD-induced obesity and metabolic disturbance, obesity-related depressive mood disorders could also be alleviated. To determine the antidepressant potential of BTS, mice fed HFD alone or HFD in combination with increasing doses of BTS were subjected to behavioral assessment. As shown in Figure 2, BTS treatment ameliorated HFD-induced depression-like behaviors. BTS at 300 mg/kg significantly reversed the increased immobility time elicited by chronic HFD intake in TST (Figure 2A). In FST (Figure 2B), BTS 300 mg/kg showed a slight, but not statistically significant, reduction in immobility time (*p* = 0.08). In OFT, HFD group showed reduced total distance traveled and center crossing numbers compared to ND. In contrast, mice receiving BTS at 300 mg/kg showed a trend towards increased total distance traveled (Figure 2C, *p* = 0.06) compared to HFD mice. However, BTS treatment did not affect center crossing numbers (Figure 2E). These behavior assessment results indicate that BTS can alleviate depression-like mood disorders induced by chronic HFD intake. In our chronic HFD-induced depression model, the positive controls FXT and SIM failed to improve HFD-induced depression-like behaviors.

### 3.3 BTS attenuates HFD-induced systemic inflammation and neuroinflammation

**FIGURE 3:**
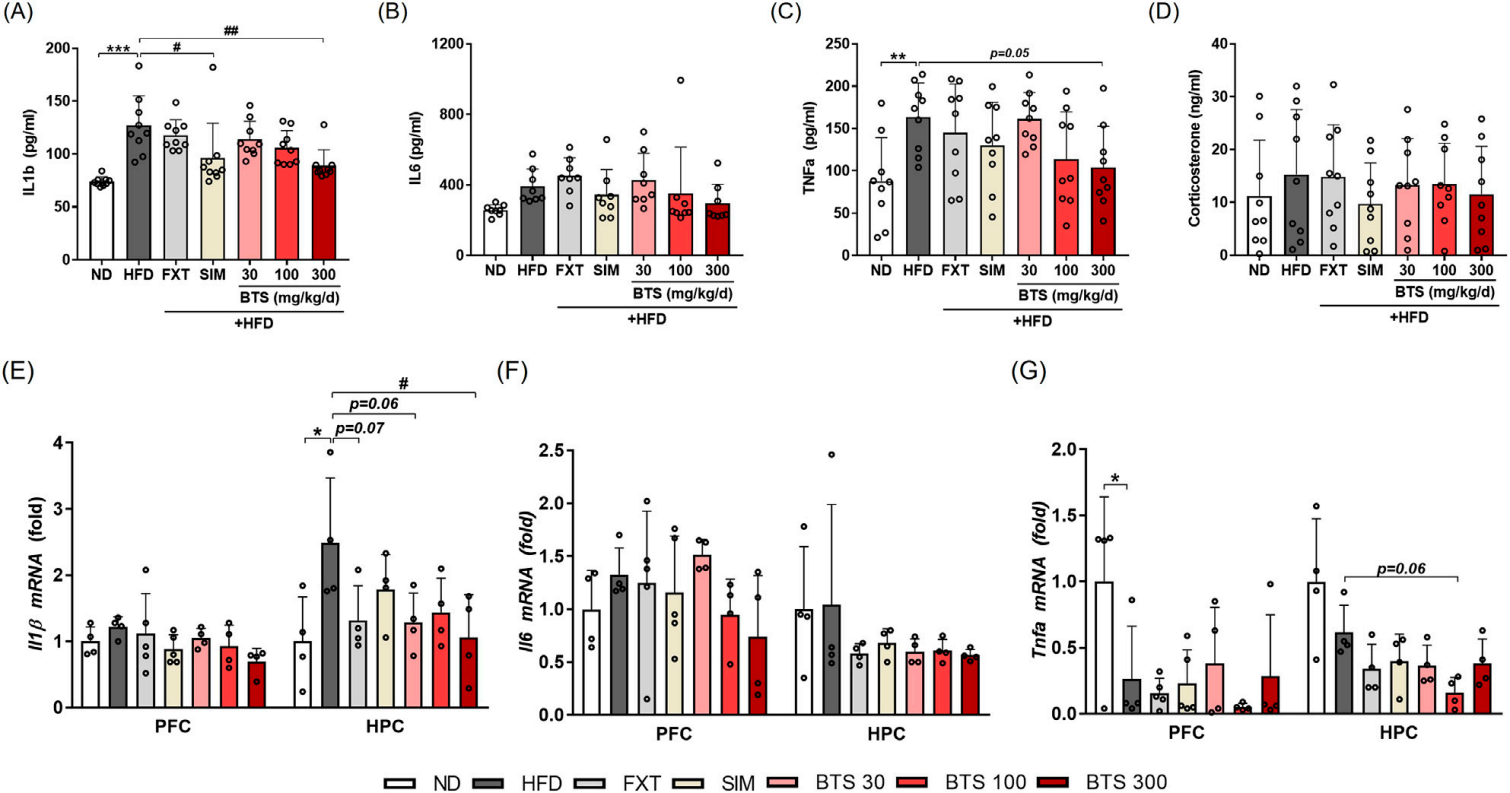
Effects of BTS on inflammatory cytokine and stress hormone expression in experimental mice with HFD-induced obesity. Following the final behavioral assessment, the mice were sacrificed, and plasma levels of pro-inflammatory cytokines (IL-1β, IL-6, and TNF-α) and corticosterone were measured using ELISA **(A–D)** (n = 8–9). In addition, mRNA expressions of pro-inflammatory cytokines (IL-1β, IL-6, and TNF-α) were measured in the PFC and HPC using qPCR **(E–G)**. Gene expression was normalized to GAPDH and compared to the ND group (n = 4–5). The values are presented as mean ± SD with individual data points; one-way ANOVA + Dunnett’s *post hoc* (control = HFD). ^*^ *p* < 0.05, ^**^ *p* < 0.01, and ^***^ *p* < 0.001 vs. ND; ^#^ *p* < 0.05, and ^##^ *p* < 0.01 vs. HFD.

Exposure to chronic HFD is known to induce intestinal barrier integrity loss, which makes it permeable to macromolecules or microbial pathogens, such as lipopolysaccharides, resulting in low-grade systemic inflammation (Rohr et al., 2020; Lama et al., 2022). Neuroinflammation induced by chronic HFD is also known to affect the hypothalamus, a control center for metabolic homeostasis, as well as extra-hypothalamic sub-brain like PFC and HPC involved in mood regulation (Guillemot-Legris and Muccioli, 2017; Lama et al., 2022). Previous studies have demonstrated that neuroinflammation in the PFC (Lorena et al., 2021; Wang T. Y. et al., 2022) or HPC (Dutheil et al., 2016; Wu et al., 2018; Park et al., 2020; Wang W. et al., 2022) areas is implicated in depression-like mood disorders. In the present study, we investigated the effect of BTS treatment on systemic inflammation and neuroinflammation in mice exposed to chronic HFD. Systemic inflammation and neuroinflammation were determined by measuring plasma levels of pro-inflammatory cytokines and gene expression in brain regions, respectively. Mice fed HFD for 10 weeks showed elevated systemic inflammation based on significantly increased plasma levels of IL-1β (Figure 3A), and TNF-α (Figure 3C) compared to ND mice. IL6 also marginally increased in the plasma of HFD control mice (Figure 3B). BTS treatment, however, alleviated the HFD-induced systemic inflammatory response in a dose-dependent manner. Specifically, BTS treatment at 300 mg/kg successfully reduced the increase in IL-1β (*p* < 0.01) and showed a borderline significant reduction in TNF-α (*p* = 0.05). SIM remarkably restored the elevated plasma levels of pro-inflammatory cytokines, especially IL-1β; however, FXT failed to alleviate systemic inflammation induced by chronic HFD. The stress hormone corticosterone (Liu et al., 2010) remained unchanged across all groups (Figure 3D). The pro-inflammatory cytokines in mouse brains exposed to chronic HFD showed region-specific expression patterns (Figures 3E–G). Compared to ND mice, marginal or significant increases in IL-1β expression were observed in both PFC and HPC, but IL-6 expression increased only marginally in PFC in HFD control mice. Unexpectedly, however, TNF-α expression was remarkably suppressed in PFC of HFD control mice. Treatment with BTS at 300 mg/kg alleviated HFD-mediated neuronal inflammation, particularly reducing IL-1β expression in HPC compared to HFD control mice. Interestingly, all pharmaceutical treatment (FXT, SIM, and BTS) in the present study intensified TNF-α suppression under chronic HFD conditions in both PFC and HPC tissues.

### 3.4 BTS restores HFD-suppressed CREB-BDNF signaling in PFC and HPC

**FIGURE 4:**
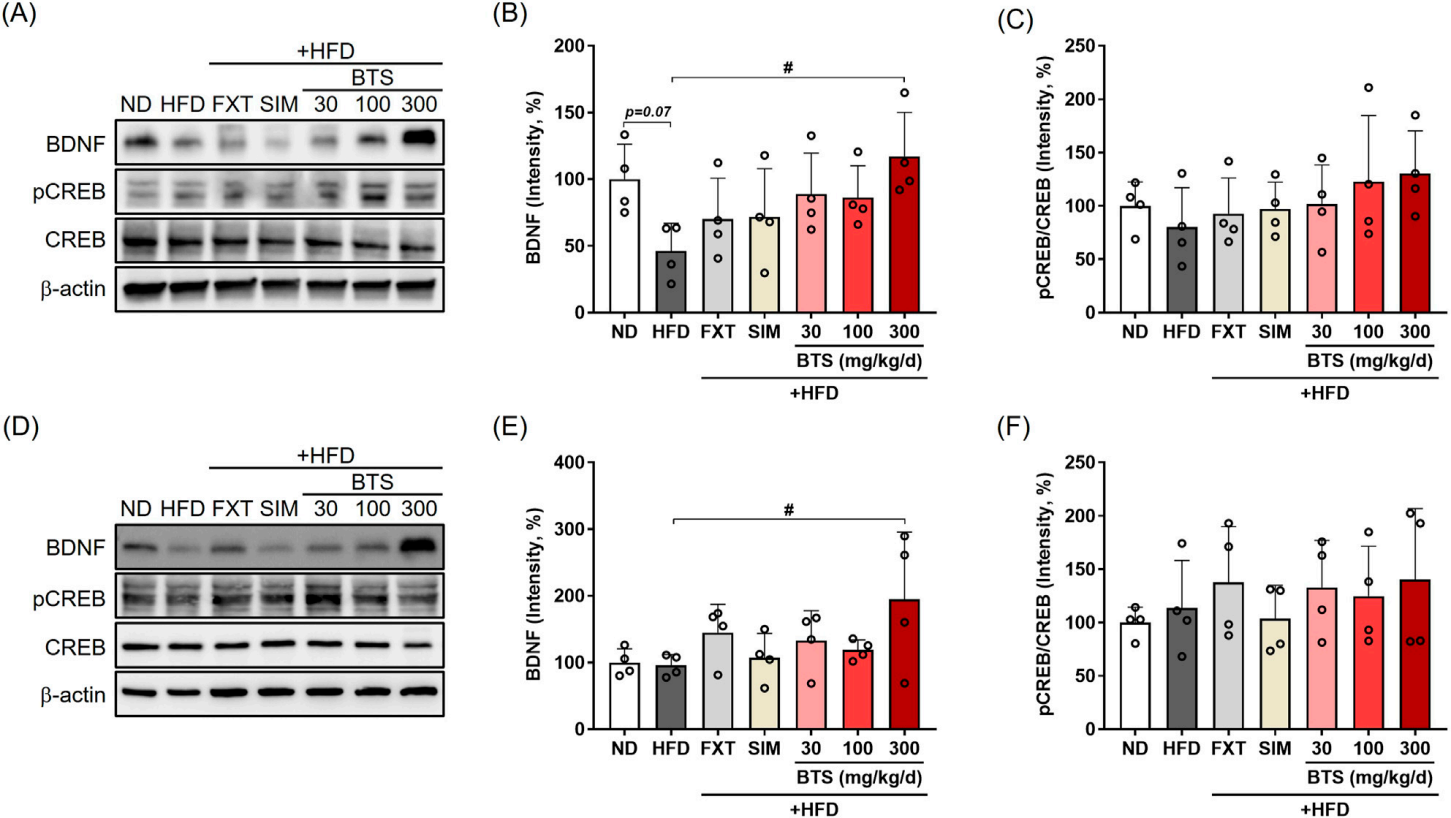
Effects of BTS on neuronal CREB-BDNF signaling in the brains of mice with HFD-induced obesity. At the end of the study, brain tissues were isolated from experimental animals and BDNF, pCREB, and total CREB expression was assessed in the PFC **(A–C)** and HPC **(D–F)** via Western blotting (n = 4). The protein expression was normalized to β-actin and compared to the ND group. The values are presented as mean ± SD with individual data points; one-way ANOVA + Dunnett’s *post hoc* (control = HFD). ^#^ *p* < 0.05 vs. HFD.

BDNF downregulation plays a key regulatory role during neuronal plasticity development and is associated with psychiatric disorders (Miranda et al., 2019). In addition to the monoamine hypothesis, increasing evidence has demonstrated CREB-BDNF signaling suppression in specific brain regions, such as the PFC, HPC, and hypothalamus, is closely related to depressive behaviors in rodent models (Vagena et al., 2019; Park et al., 2020; Ji et al., 2022; Youssef et al., 2022). Thus, CREB-BDNF signaling is one of the most attractive pharmaceutical targets for treating depression (Ji et al., 2022). Therefore, we determined the changes in CREB-BDNF signaling in the PFC and HPC of mice exposed to chronic HFD in combination with pharmacological interventions. Activation of CREB-BDNF signaling in the PFC and HPC was determined by monitoring CREB phosphorylation at Ser133 via Western blotting. Chronic HFD intake suppressed BDNF expression in PFC by 53.9%, along with decreased CREB phosphorylation (Figures 4A–C). BTS treatment enhanced CREB-BDNF signaling and restored the decreased BDNF expression in a dose-dependent manner. BDNF expression in the PFC of mice treated with 300 mg/kg BTS was comparable to that of ND mice. Although CREB-BDNF signaling suppression under chronic HFD was not as dramatic in HPC tissue, mice treated with BTS showed enhanced CREB-BDNF signaling, with BDNF expression increased by 103.4% at 300 mg/kg BTS (Figures 4D–F). Treatment with FXT or SIM marginally activated CREB-BDNF signaling in PFC and HPC tissues but not to the extent observed with BTS.

### 3.5 BTS differentially regulates NMDA receptor subunit expression in PFC and HPC

**FIGURE 5:**
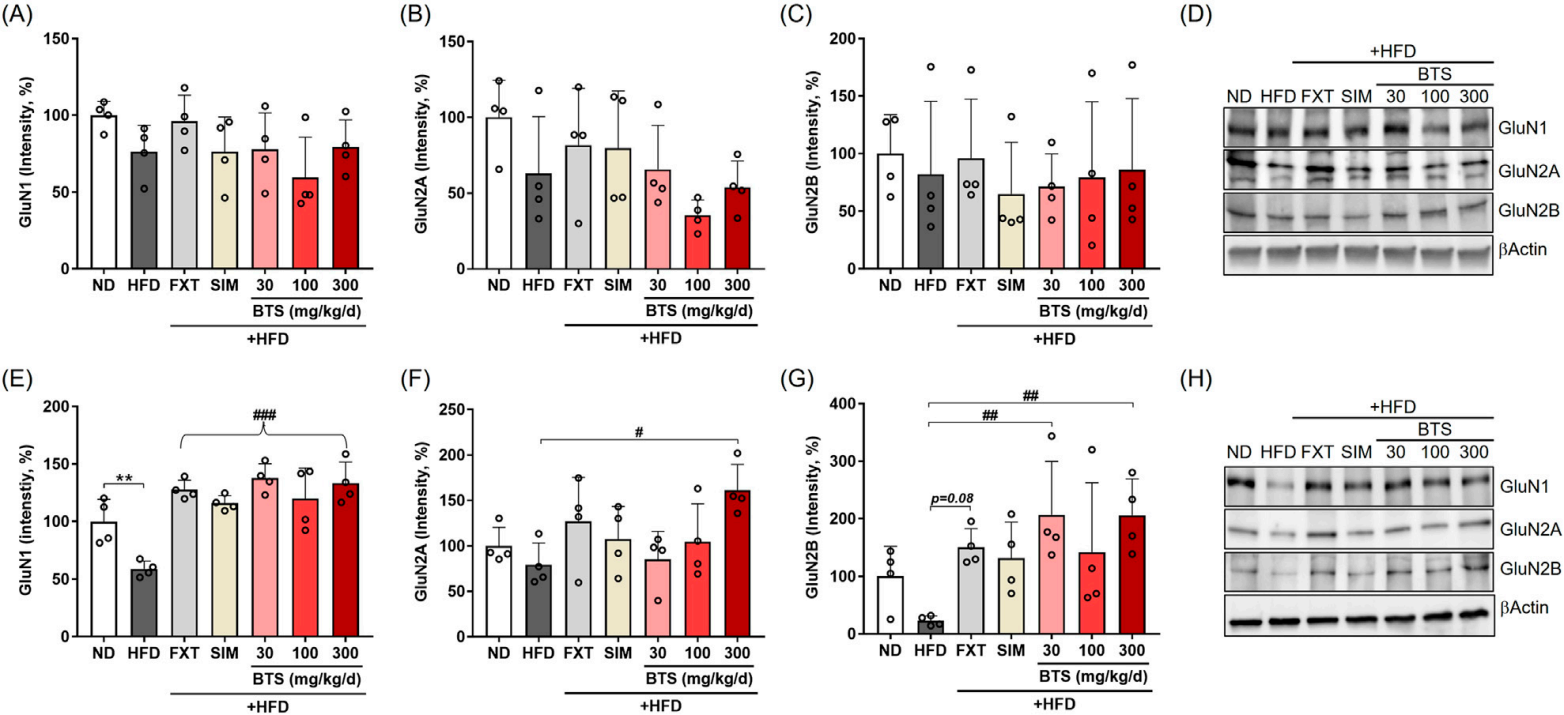
Effects of BTS on the expression levels of NMDA receptor subtypes in the brains of mice with HFD-induced obesity. GluN1, GluN2A, and GluN2B expression were assessed in the PFC **(A–D)** and HPC **(E–H)** via Western blotting (n = 4). The protein expression was normalized to β-actin and compared to the ND group. The values are presented as mean ± SD with individual data points; one-way ANOVA + Dunnett’s *post hoc* (control = HFD). ^*^ *p* < 0.05 and ^**^ *p* < 0.01 vs. ND; ^#^ *p* < 0.05, ^##^ *p* < 0.01, and ^###^ *p* < 0.001 vs. HFD. (F) The curly bracket denotes that all drug-treated groups showed a significant increase relative to HFD group.

Since the non-competitive NMDA receptor antagonist ketamine exhibits rapid and long-lasting antidepressant potential at a sub-anesthetic dose in both clinical and non-clinical studies, the NMDA receptor has been suggested as a novel pharmaceutical target for treating depression (Vollenweider and Kometer, 2010; Lindholm et al., 2012; Weston et al., 2021). In the present study, we investigated changes in NMDA receptor subtype expression in the PFC and HPC following chronic HFD intake in combination with pharmaceutical interventions. In the PFC of HFD control mice, GluN1 and GluN2A expression decreased by 23.8% and 37.1% compared to ND mice, respectively (Figures 5A–D). Interestingly, BTS treatment further downregulated GluN1 (40.5% at 100 mg/kg) and GluN2A (64.6% at 100 mg/kg) expression. FXT and SIM treatment marginally reduced HFD-induced downregulation of some NMDA receptors but did not reach statistical significance. However, in HPC tissues (Figures 5E–H), GluN1 was significantly downregulated in HFD control mice, and GluN2B expression showed a tendency to decrease compared to ND. BTS treatment successfully reversed the downregulation of NMDA receptors. Specifically, BTS at 300 mg/kg increased GluN1 by 126.5%, GluN2A by 103.4%, and Glu2B by 802.1% compared to HFD control mice. Significant increases in GluN1 expression levels were observed in all drug treatment groups compared to HFD group. Both FXT and SIM treatment could reverse the HFD-induced downregulation of GluN1 (FXT by 116.6%; SIM by 96.8%, *p* < 0.001 respectively) and GluN2B (FXT by 561.0%, *p* = 0.08) in HPC. At the transcriptional level, NMDA receptors were regulated differently according to brain region. No significant changes in NMDA receptor expressions were observed in either the PFC or HPC following chronic HFD intake. However, all pharmacological interventions, including BTS, enhanced the expression of NMDA receptor subtypes such as GluN1 and GluN2A in HPC tissues (Supplementary Figure S2).

### 3.6 BTS modulates 5-HT signaling pathways in PFC and HPC

**FIGURE 6:**
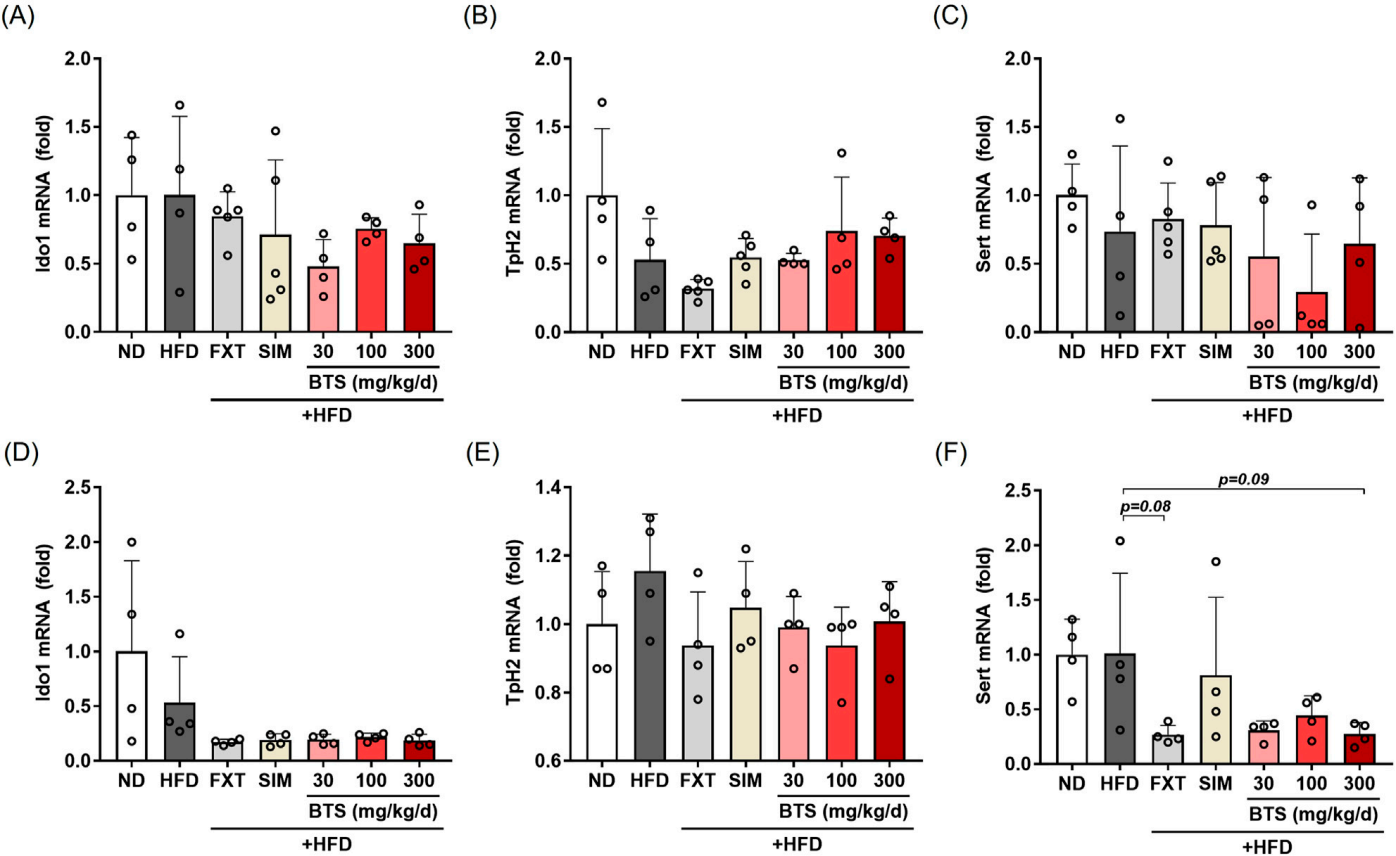
Effects of BTS on gene expression related to 5-HT signaling in experimental mice with HFD-induced obesity. mRNA expression of Ido1, Tph2, and SERT were assessed in the PFC **(A–C)** and HPC **(D–F)** using qPCR. Gene expression was normalized to GAPDH and compared to the ND group (n = 4–5). The values are presented as mean ± SD with individual data points; one-way ANOVA + Dunnett’s *post hoc* (control = HFD).

Brain 5-HT is a neurotransmitter that plays a key role in a variety of psychiatric behaviors, including depression (Best et al., 2010). In serotonergic neurons, L-tryptophan (Trp) is involved in 5-HT synthesis, whose rate is limited by Trp availability (Hoglund et al., 2019). The majority of Trp is converted to kynurenic acid and quinolinic acid, rather than 5-HT, by indoleamine 2,3-dioxygenase 1 (IDO1), the first rate-limiting enzyme of the kynurenine pathway in extrahepatic tissues (Kwidzinski and Bechmann, 2007). A portion of Trp is converted to 5-HT by neuronal tryptophan hydroxylase 2 (TpH2), the rate-limiting enzyme in the 5-HT synthesis pathway (Hoglund et al., 2019). Therefore, the intracellular 5-HT level depends on the equilibrium balance between these two metabolic pathways. In addition to the Trp-metabolizing pathways, the synaptic 5-HT level is regulated by a membrane serotonin transporter (SERT) that reuptakes 5-HT released into the synaptic cleft from the presynaptic 5-HT vesicles; SERT is a molecular target for SSRI antidepressant drugs (Kulikov et al., 2018). In the present study, we investigated the effect of BTS on IDO1, TpH2, and SERT expression in mice exposed to a chronic HFD by measuring their intracellular mRNA levels. In PFC tissues, IDO1 mRNA showed no significant change (Figure 6A). A marginal reduction in TpH2 expression (47% decrease) was observed in PFC tissues (Figure 6B), which was partially recovered by BTS treatment at 100 and 300 mg/kg. A partial decrease in SERT expression was observed in HFD mice treated with BTS 100 mg/kg (Figure 6C). Interestingly, FXT treatment appeared to further downregulate TpH2 expression in PFC. In HPC tissues (Figures 6D–F), chronic HFD intake marginally suppressed IDO1 expression (47% decrease). Furthermore, all pharmacological interventions, including BTS, further decreased IDO1 expression in HFD mice (not significance). SERT expression in HPC showed a slight, but not statistically significant, reduction with BTS 300 mg/kg (*p* = 0.09) or FXT (*p* = 0.08) co-administration with chronic HFD. No remarkable changes in TpH2 expression were observed in HPC tissues following chronic HFD intake or pharmaceutical interventions.

### 3.7 BTS restores brain 5-HT levels in HFD-induced mice

**FIGURE 7:**
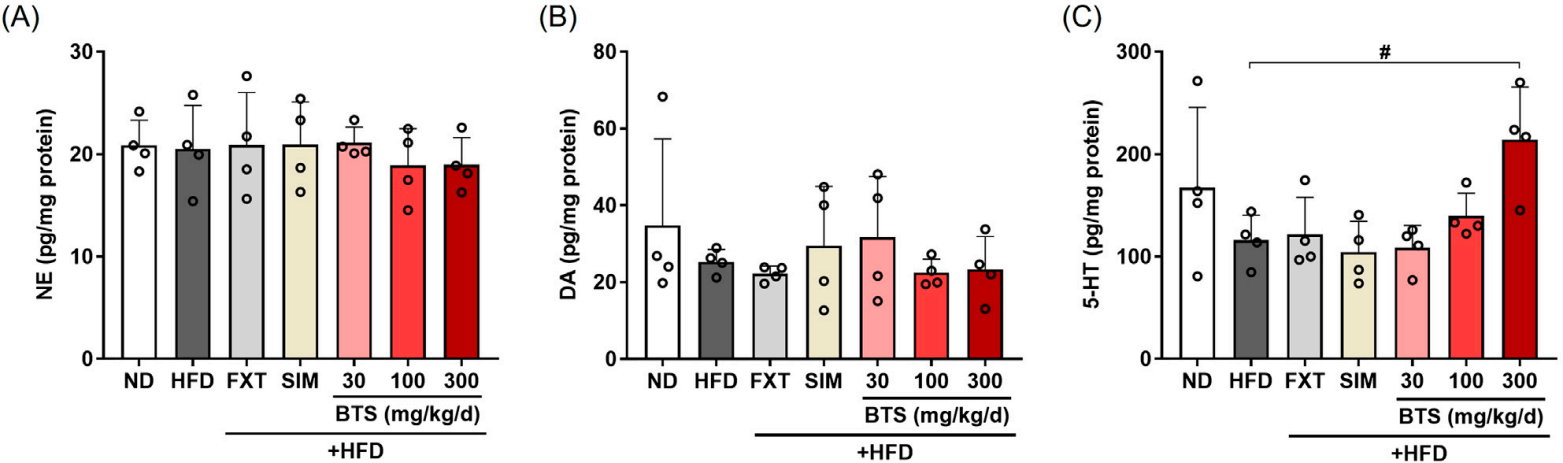
Effects of BTS on monoamine levels in the brains of mice with HFD-induced obesity. NE **(A)**, DA **(B)**, and 5-HT **(C)** levels in whole brain lysates were determined using ELISA (n = 4). The values are presented as mean ± SD with individual data points; one-way ANOVA + Dunnett’s *post hoc* (control = HFD). ^#^ *p* < 0.05 vs. HFD.

The monoamine hypothesis states that the depletion of monoamines, such as NE, DA, and 5-HT, in the central nervous system is the underlying pathophysiological basis of depression (Delgado, 2000; Tiemeier, 2003). This hypothesis is supported by the clinical improvement of depression by agents that increase the synaptic concentrations of monoamines (Hirschfeld, 2000). In previous studies, experimental animals subjected to chronic stressful conditions (Oh et al., 2018; Nazeem et al., 2021) or depression-inducing drugs (Yu et al., 2016; Park et al., 2020) have shown decreased monoamine levels in the brain. In contrast, another research group reported elevated monoamine levels in the amygdala of a mouse model (Lama et al., 2022). In the present study, we investigated the effect of HFD alone or in combination with pharmaceutical interventions on monoamine levels in the whole brain. As shown in Figure 7, no significant changes in brain NE were observed with either treatment. Conversely, marginal decreases in DA (27% decrease) and 5-HT levels (30.5% decrease) were observed in HFD control mouse brains compared to ND mice. BTS treatment increased brain 5-HT levels, and its level in mice receiving 300 mg/kg BTS was comparable to that in ND mice. In our HFD-induced depression model, FXT and SIM failed to restore the decreased levels of DA and 5-HT in the entire brain.

## 4 Discussion

Previous studies have demonstrated the anti-obesity effects of BTS. In HFD animal models, BTS administration decreased adipose tissue weight and the plasma levels of TCHO, TG, and free fatty acids (Kobayashi et al., 2017; Saeki et al., 2024). Clinically, a randomized trial in obese women with impaired glucose tolerance showed loss of body weight and abdominal visceral fat without changing the adjusted resting metabolic rate (Hioki et al., 2004). These data suggest that BTS holds therapeutic potential to treat obesity and obesity-associated diseases. Against this background, evidence regarding whether BTS modulates mood disorders that co-occur with obesity has remained limited. Here, we evaluated the effect of BTS on depressive behaviors in experimental animals with HFD-induced obesity and identified the mechanism underlying its antidepressant potential. Chronic continuous HFD provoked concurrent dysmetabolism and depressive-like phenotypes, whereas BTS improved both metabolic dysfunction and depressive behaviors, while attenuating neuroinflammation, restoring BDNF signaling, and normalizing hippocampal NMDA-receptor and 5-HT signaling. We next interpret these findings within mechanistic frameworks linking obesity and depression.

Increasing evidence demonstrates that chronic low-grade systemic inflammation is associated with obesity and promotes susceptibility to depression by disrupting several neuronal functions, including neurotransmitter synthesis, reuptake, and release (Ellulu et al., 2017; Beurel et al., 2020). In line with previous studies, our HFD mice showed elevated inflammatory markers along with depressive-like behaviors. Notably, repeated administration of BTS up to 300 mg/kg, at non-toxic doses, more effectively reduced HFD-induced inflammation and behavioral despair than FXT or SIM, aligning with BTS’s reported metabolic/anti-inflammatory effects (e.g., reduced adiposity, improved glycemic/lipid profiles, and suppressed adipose inflammation) that provide pharmacological plausibility for our observations (Hioki et al., 2004; Fujisaka et al., 2020).

In addition to anti-inflammatory effect of BTS, it enhanced BDNF expression in PFC and HPC, consistent with clinical and preclinical evidence linking BDNF regulation to antidepressant efficacy (He et al., 2025; Li et al., 2025). This BDNF enhancement may contribute to both mood improvement and metabolic benefits observed in our study. BDNF is implicated in metabolic regulation, with central infusion leading to improved glycemic control and antidepressant effects in obese diabetic mice (McMurphy et al., 2019). This provides a consistent link to the concurrent metabolic benefits we observed.

Neuroplasticity modulates neural circuits that control mood disorders, providing a new perspective on antidepressants and mood stabilizers related to neuroplasticity (Du et al., 2004). NMDA receptors are crucial mediators of synaptic transmission and plasticity in the central nervous system (Niciu et al., 2012; Paoletti et al., 2013). BDNF plays a critical role in regulating the expression, activity, and trafficking of NMDARs at synapses, and these regulatory effects of BDNF signaling are essential for NMDAR-mediated LTP and LTD, which underlie synaptic function and plasticity (Ninan et al., 2010; Afonso et al., 2019; De Luca et al., 2024). Consistent with evidence that dentate gyrus microinjection of an NMDAR antagonist reversed the BDNF reduction while improving spatial memory in obese rats (Lv et al., 2024), our finding that BTS restored hippocampal BDNF and NMDAR subunits supports a mechanistic link to NMDAR-dependent plasticity under HFD stress.

Our findings demonstrate alterations in the serotonergic system, including abnormalities in SERT protein expression and whole brain 5-HT levels. These findings are consistent with those of several preclinical studies. Hu et al. reported that hippocampal SERT and Tph2 expression were downregulated in HFD-fed mice, leading to serotonergic neurotransmission impairment and depressive-like behavior, which were alleviated by an IOD1 inhibitor (Hu et al., 2024). Similarly, SERT-deficient mice fed a Western-style diet exhibited impaired brain insulin tolerance and abnormal expression of 5-HT receptor subtypes (Veniaminova et al., 2020; Anthony et al., 2024), suggesting that serotonergic deficiency may exacerbate both metabolic- and mood disorder-related phenotypes. In the present study, after 10-week continuous HFD, whole brain 5-HT levels showed a modest, though not statistically significant, decrease. However, BTS treatment effectively restored these levels. Notably, the mechanism underlying this serotonin increase could be identified through examination of SERT expression changes in the HPC. Although not reaching statistical significance, BTS at 300 mg/kg demonstrated a trend toward reduction in SERT expression. Based on these findings, we propose that BTS serves as a promising therapeutic alternative that can alleviate obesity-depression comorbidity through SSRI-like mechanisms.

Building on these mechanistic insights, our findings demonstrate that BTS functions as a multi-target therapeutic agent that modulates obesity-depression comorbidity by mediating anti-inflammatory pathways, BDNF-NMDAR signaling, and serotonergic pathways in brain. Importantly, obesity-related depression is etiologically heterogeneous. Immune, metabolic, and synaptic dysfunctions interact together, and behavioral improvement likely requires simultaneous modulation of multiple networks rather than maximal modulation of a single network (Kaufmann et al., 2024). By distributing pharmacological effects across these networks, a multi-target agent can achieve comparable or superior efficacy without excessive dose escalation on any one mechanism, potentially reducing mechanism-specific adverse effects. In our long-term oral paradigm, SIM (conventional anti-obesity agent) and FXT (SSRI class of antidepressants) were less effective than BTS on depressive-like behaviors. This observation was not fully consistent with previous reports in which SIM ameliorated HFD-induced emotional disorders by improving cannabinoid analogs (Wang et al., 2017) or by reducing HPC inflammation (Wu et al., 2019). This discrepancy in the pharmacological efficacy of SIM might originate from different drug dosing strategies and experimental context. In previous studies, SIM was administered to mice in the middle of the HFD period for ∼4 weeks at 5–10 mg/kg i.p., whereas we administered SIM throughout the HFD period. Prolonged SIM has a higher risk of adverse metabolic effects and can affect hippocampal cholesterol homeostasis with cognitive consequences (Schultz et al., 2018; Na et al., 2020). Consistent with this context, under our chronic paradigm SIM exerted only weak effects on depressive-like behaviors, slightly improving HFD-induced abnormal metabolic states, and pro-inflammatory cytokines. Similarly, FXT showed limited therapeutic effects in our HFD-induced obesity model. Long-term SSRI use had limited appetite suppression effects (Mouawad et al., 2025). Additionally, most participants showed decrease antidepressant effects during prolonged SSRI monotherapy (Katz, 2011). In summary, our comparative analysis reveals fundamental limitations of conventional single-target approaches. These comparative findings underscore the paradigm shift needed from conventional monotherapy to multi-target approaches, positioning BTS as a promising therapeutic strategy for the complex pathophysiology underlying obesity-depression comorbidity.

While these findings demonstrate BTS’s therapeutic potential, several methodological limitations should be considered. First, neurotransmitter levels were measured in whole-brain homogenates rather than in specific regions. This approach allowed for the interpretation of network-level changes, but it did not elucidate direct correlations between behavioral outcomes and region-specific neurochemical changes. Future studies should employ regional analysis approaches (e.g., LC-MS/MS analysis in discrete brain areas) to provide clearer insights into the spatial dynamics of BTS action. Second, our molecular profiling focused primarily on a targeted panel of pro-inflammatory mediators, neuroplasticity-related markers and monoaminergic regulators, which limits our understanding of inter-pathway interactions and upstream regulatory mechanisms that could mediate BTS’s multi-target effects. Future work should adopt broader analytical approaches and metabolomics to better delineate the coordinated networks underlying BTS action. Addressing these methodological considerations will be crucial for translating BTS into a clinically viable therapeutic intervention for obesity-depression comorbidity.

Finally, although BTS demonstrated favorable tolerability in our animal study and effectively ameliorated obesity-related depressive phenotypes through multi-target mechanisms, the clinical safety profile of BTS remains to be fully established. Rare adverse events such as drug-induced liver injury have been reported in the Japanese Adverse Drug Event Report (JADER) database (Ishida et al., 2022), underscoring the importance of systematic safety monitoring in clinical settings. As our present work was limited to preclinical models and mechanistic validation, future research should prioritize well-designed clinical trials to confirm efficacy, optimize dosing strategies, and carefully evaluate potential adverse effects in human populations. Such efforts will be crucial to translate the promising preclinical findings of BTS into a clinically applicable therapeutic strategy for patients with obesity-related depression.

## 5 Conclusion

In this study, we demonstrated that BTS mitigated depression-like behaviors in HFD-induced obese mice, mediated in part through the regulation of systemic/neuroinflammation, BDNF signaling, hippocampal NMDARs, and intrasynaptic 5-HT levels. Additionally, our results suggest that chronic BTS treatment has potential advantages over FXT and SIM, consistent with its multi-target effects across immune, neuroplastic, and monoaminergic system. The simultaneous therapeutic effects of BTS on depression and obesity may represent a new paradigm for treating these comorbidities and could play a significant role in clinical research and the establishment of evidence-based treatment guidelines.

## Data Availability

The original contributions presented in the study are included in the article/Supplementary Material, further inquiries can be directed to the corresponding author.

## Abbreviations

HFD, high-fat diet; 5-HT, serotonin; HPC, hippocampus; SSRI, selective 5-HT reuptake inhibitor; BTS, Bangpungtongsung-san; TCHO, total cholesterol; IHFD, intermittent HFD; PBS, phosphate-buffered saline; ND, normal diet; FXT, fluoxetine; SIM, simvastatin; OFT, open-field test; TST, tail suspension test; FST, forced swimming test; PFC, prefrontal cortex; BDNF, brain-derived neurotrophic factor; CREB, cAMP response element-binding protein; pCREB, phospho-CREB; NMDA, N-methyl-D-aspartic acid; GluN, NMDA receptor; qPCR, quantitative real-time chain reaction; DA, dopamine; NE, norepinephrine; TNF-α, tumor necrosis factor-alpha; IL, interleukin; TG, triglyceride; HDL-C, high-density lipoprotein cholesterol; Trp, L-tryptophan; IDO1, indoleamine 2,3-dioxygenase 1; TpH2, tryptophan hydroxylase 2; SERT, serotonin transporter.
