# Supplementary figures and images for: Bangpungtongsung-san alleviates depressive-like behavior and metabolic disturbances in high-fat diet-induced obesity: mechanisms involving inflammation, CREB/BDNF signaling, and NMDA receptor modulation
Source: Front Pharmacol. 2025 Oct 30;16:1565592. doi: 10.3389/fphar.2025.1565592 (PMC12611972; doi:10.3389/fphar.2025.1565592)

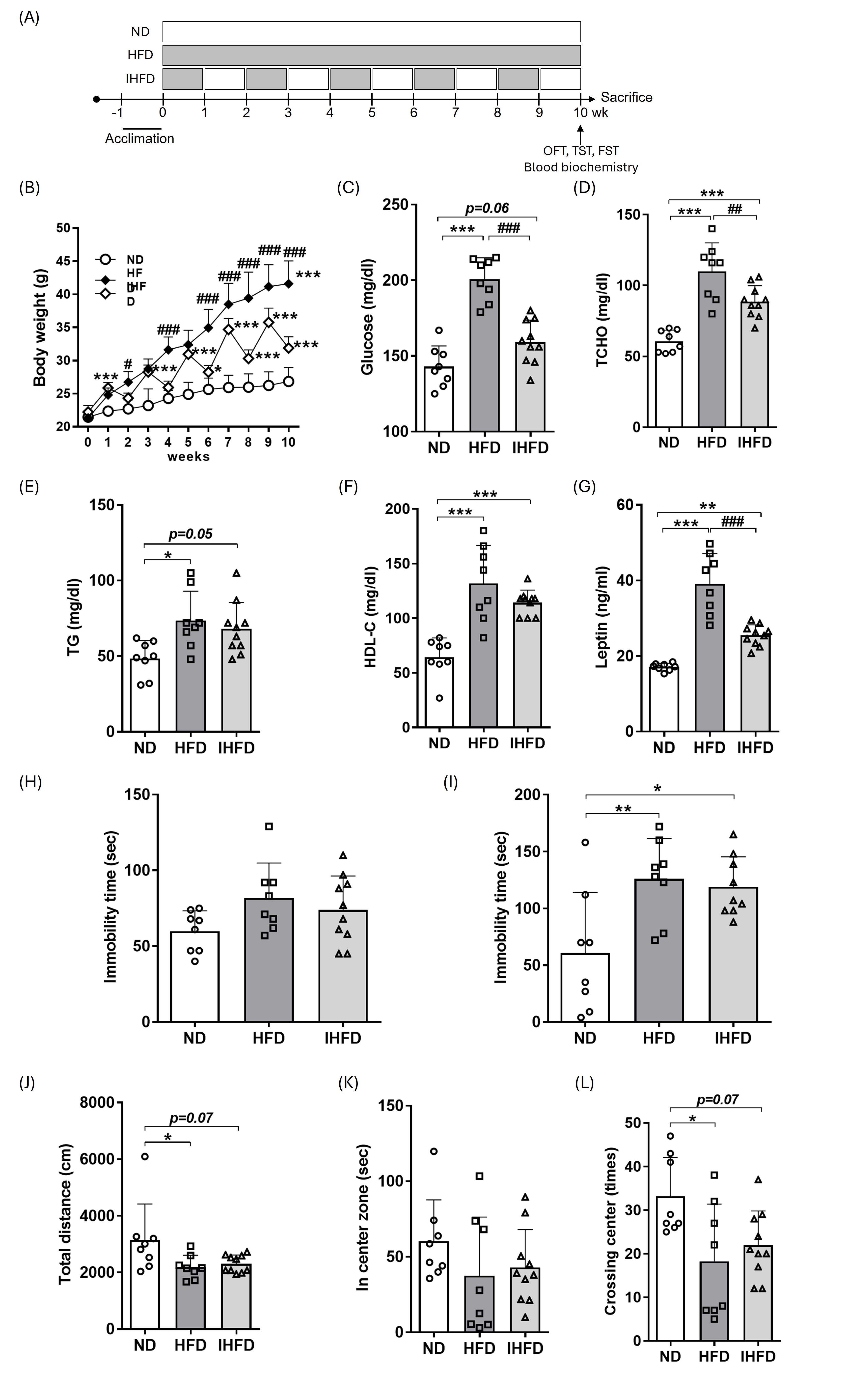

Supplement: Supplementary file 1 [file Image1.jpeg]

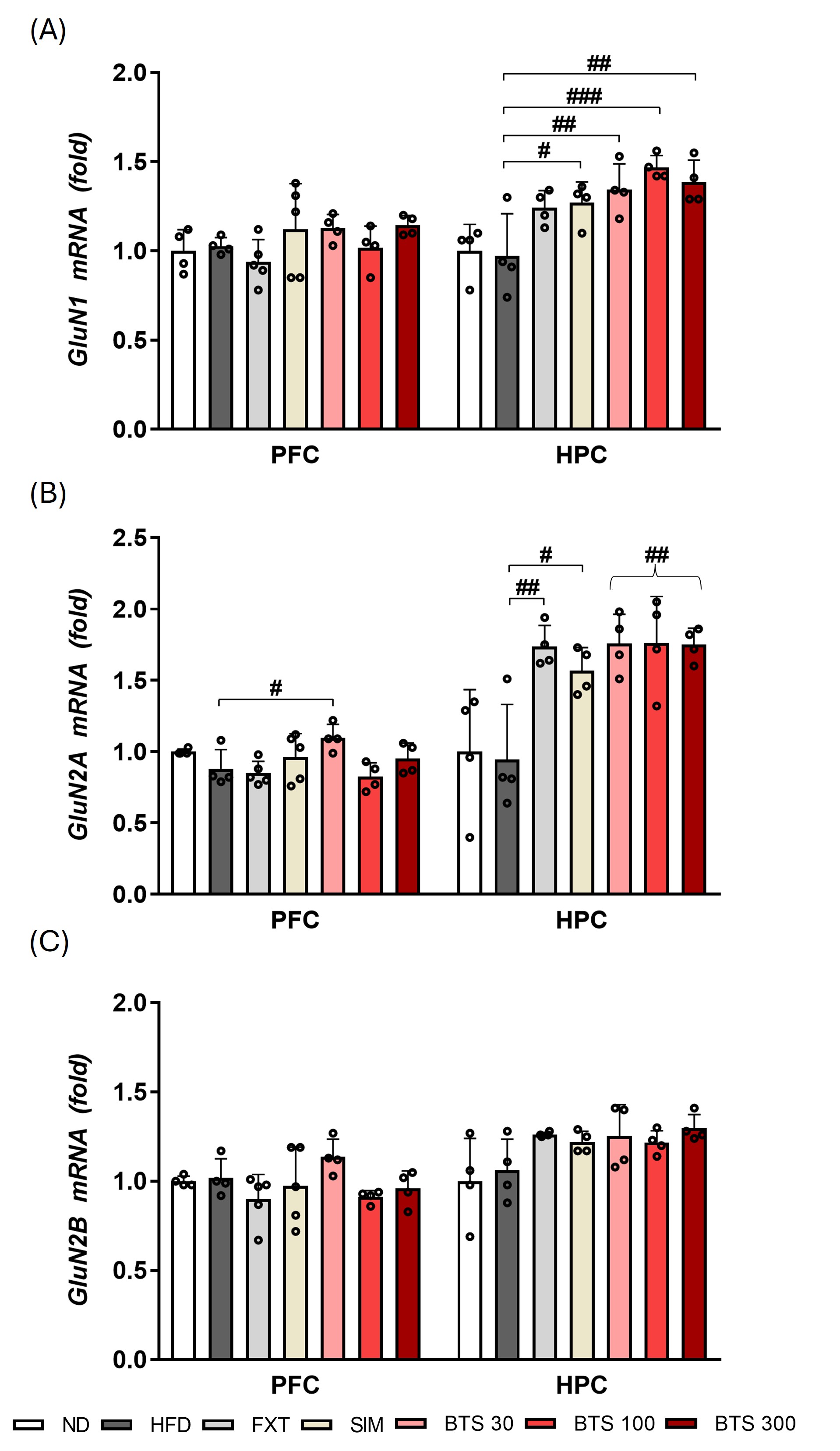

Supplement: Supplementary file 2 [file Image2.jpeg]
