# Supplementary material for: Bangpungtongsung-san alleviates depressive-like behavior and metabolic disturbances in high-fat diet-induced obesity: mechanisms involving inflammation, CREB/BDNF signaling, and NMDA receptor modulation
Source: Front Pharmacol. 2025 Oct 30;16:1565592. doi: 10.3389/fphar.2025.1565592 (PMC12611972; doi:10.3389/fphar.2025.1565592)
Supplement: Supplementary file 3 [file DataSheet1.docx]

***Supplementary materials***

**Supplementary Table 1. Content of marker compounds in BTS extract and corresponding daily doses.**

| Compound | Content (mg/g extract) | 30 mg/kg/day | 100 mg/kg/day | 300 mg/kg/day |
| --- | --- | --- | --- | --- |
| Gallic acid | 1.44 | 0.0432 | 0.144 | 0.432 |
| Geniposide | 2.66 | 0.0798 | 0.266 | 0.798 |
| Albiflorin | 1.87 | 0.0561 | 0.187 | 0.561 |
| Paeoniflorin | 1.98 | 0.0594 | 0.198 | 0.594 |
| Liquiritin apioside | 0.76 | 0.0228 | 0.076 | 0.228 |
| Liquiritin | 1.20 | 0.036 | 0.12 | 0.36 |
| Nodakenin | 0.74 | 0.0222 | 0.074 | 0.222 |
| Benzoic acid | 1.43 | 0.0429 | 0.143 | 0.429 |
| Baicalin | 11.20 | 0.336 | 1.12 | 3.36 |
| Wogonoside | 1.53 | 0.0459 | 0.153 | 0.459 |
| Glycyrrhizin | 2.02 | 0.0606 | 0.202 | 0.606 |

**
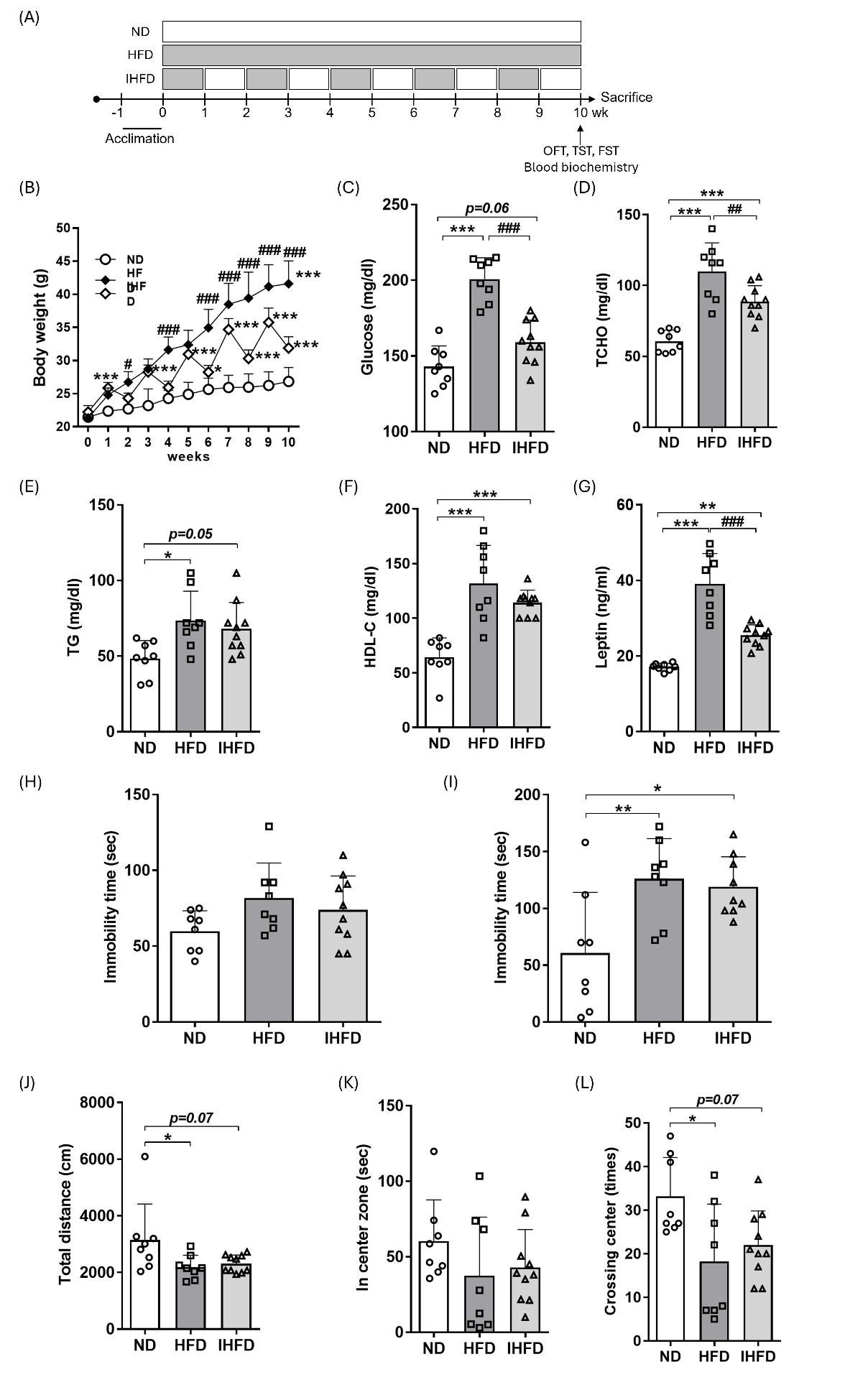
**

**Supplementary Figure S1.** Effects of HFD feeding strategy on body weight, metabolic states and depressive-like behaviors in experimental mice. Male C57BL/6N mice were orally fed ND, HFD, or IHFD every day for 10 weeks. (A) Schematic diagram of diet schedule and behavioral assessments (n = 8-10). (B) Body weights were recorded every week throughout the experimental period. The values are presented as mean ± SD; two-way ANOVA + Tukey’s post hoc. Fasting glucose (C), TCHO (D), TG (E), HDL-C (F), and leptin (G) were analyzed from plasma collected at the experimental endpoint. On 10th week of experiment, mice were performed behavioral tests in the following order; OFT, TST, and FST. Immobility times in the TST (H) and FST (I), total traveling distance (J), time spent in the center zone (K), and the number of center crosses (L) in the OFT of individual animals were measured at the end of the study. The values are presented as mean ± SD with individual data points; one-way ANOVA + Tukey’s post hoc.


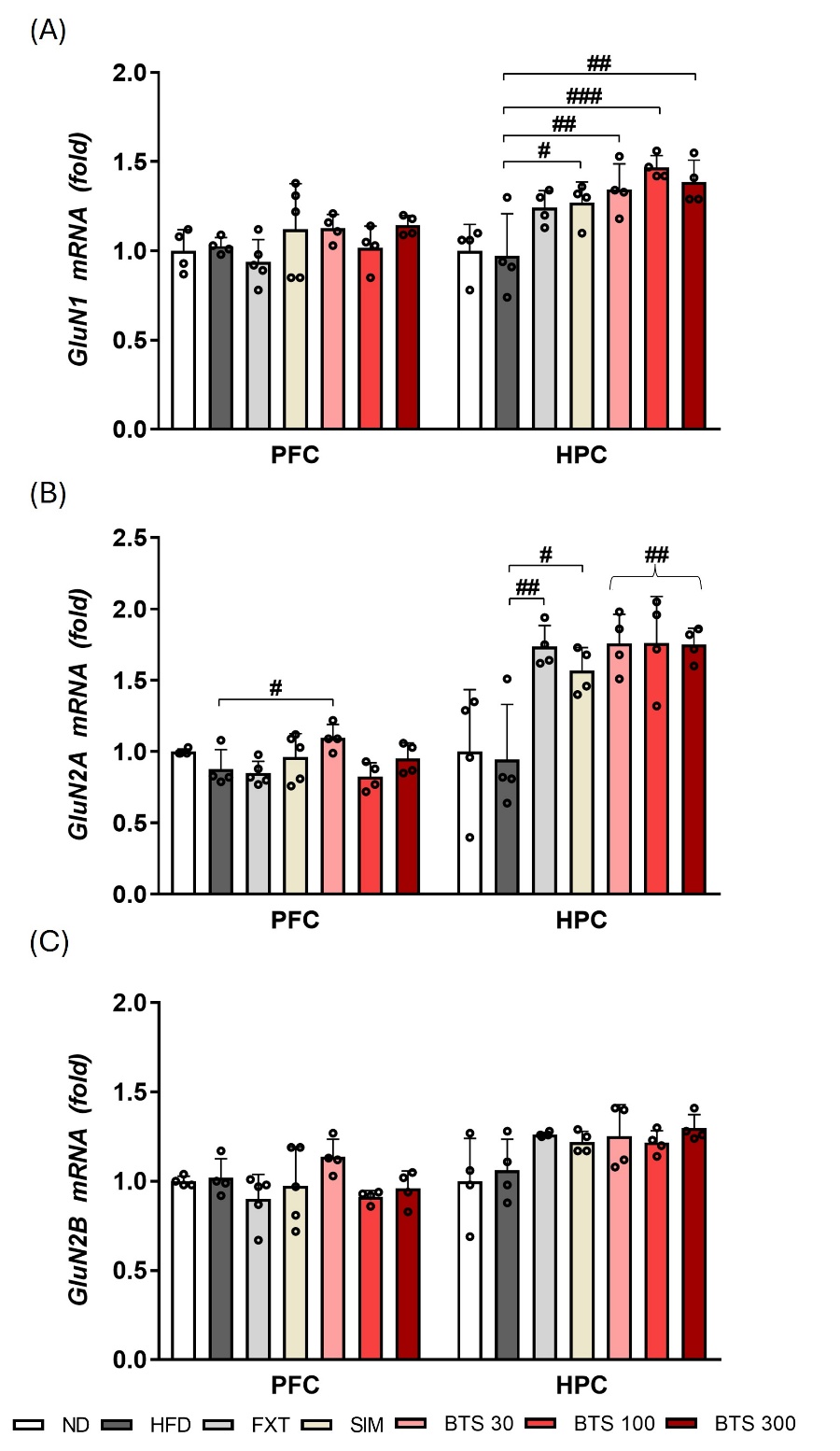


**Supplementary Figure S2.** Effects of BTS on mRNA expression of NMDA receptor subtypes in mouse brains with HFD-induced obesity. At the end of study, brain tissues were isolated and mRNA expression of GluN1, GluN2A and GluN2B were determined in the PFC and HPC by qPCR. The gene expression was normalized to GAPDH and compared to the ND group. The values are presented as mean ± SD with individual data points; one-way ANOVA + Dunnett’s post hoc (control = HFD). ^#^*p* < 0.05, ^##^*p* < 0.01, and ^###^*p* < 0.001 *vs*. HFD. (B) The curly bracket denotes that all drug-treated groups showed a significant increase relative to HFD group.
